# Age-modified factors associated with placental malaria in rural Burkina Faso

**DOI:** 10.1186/s12884-022-04568-4

**Published:** 2022-03-24

**Authors:** Biébo Bihoun, Serge Henri Zango, Maminata Traoré-Coulibaly, Innocent Valea, Raffaella Ravinetto, Jean Pierre Van Geertruyden, Umberto D’Alessandro, Halidou Tinto, Annie Robert

**Affiliations:** 1 Unité de recherche clinique de Nanoro, Institut de recherche en science de la santé, Nanoro, Burkina Faso; 2 Pôle Epidémiologie et Biostatistiques, Institut de Recherche Expérimentale et Clinique, Université catholique de Louvain, Brussels, Belgium; 3 Public Health Department, Institute of Tropical Medicine, Antwerp, Belgium; 4 Global Health Institute, University of Antwerp, Antwerp, Belgium; 5 MRC Unit The Gambia at the London School of Hygiene and Tropical Medicine, Fajara, Gambia

**Keywords:** Pregnancy, Placenta, Malaria, Risk factors, Burkina Faso

## Abstract

**Background:**

Malaria in pregnancy can result in placental infection with fetal implications. This study aimed at assessing placental malaria (PM) prevalence and its associated factors in a cohort of pregnant women with peripheral malaria and their offspring.

**Method:**

The data were collected in the framework of a clinical trial on treatments for malaria in pregnant women . Placental malaria (PM) was diagnosed by histopathological detection of parasites and/or malaria pigment on placenta biopsies taken at delivery. Factors associated with PM were assessed using logistic regression.

**Results:**

Out of 745 biopsies examined, PM was diagnosed in 86.8 % of women. Acute, chronic and past PM were retrieved in 11 (1.5 %), 170 (22.8 %), and 466 (62.6 %) women, respectively. A modifying effect was observed in the association of gravidity or anemia at the study start with pooled PM (presence of parasites and/or malaria pigment). In women under 30, gravidity ≤ 2 was associated with an increased prevalence of pooled PM but in women aged 30 years or more, gravidity was no more associated with pooled PM (OR 6.81, 95 % CI 3.18 – 14.60; and OR 0.52, 95 % CI 0.10 – 2.76, respectively). Anemia was associated with pooled PM in women under 30 (OR 1.96, 95 % CI 1.03 – 3.72) but not in women aged 30 years or more (OR 0.68, 95 % CI 0.31 – 1.49). Similarly, the association of gravidity with past-chronic PM depended also on age. A higher prevalence of active PM was observed in women under 30 presenting with symptomatic malaria (OR 3.79, 95 % CI 1.55 – 9.27), while there was no significant increase in the prevalence of active PM (presence of parasites only) in women with symptomatic malaria when aged 30 years or more (OR 0.42, 95 % CI 0.10 – 1.75). In women with chronic PM, the prevalence of low birth weight or prematurity was the highest (31.2 %) as compared with past PM or no PM.

**Conclusion:**

Despite the rapid diagnosis and efficacious treatment of peripheral infection, the prevalence of placental malaria remained high in women with *P. falciparum* peripheral infection in Nanoro, especially in younger women This underlines the importance of preventive measures in this specific group.

## Background

In 2019, 12 million pregnant women were infected by malaria worldwide. According to World Health Organization, more than one-third (35 %) of these infections occurred in the West-African region [1]. *Plasmodium falciparum* (*P.falciparum*) is the parasite responsible for a large majority of cases of malaria in the general population in Africa [2]. Despite the endemicity of malaria in sub-Saharan African countries, the prevalence of P.falciparum infection following microscopic examination of the peripheral blood of women with prolonged duration of pregnancy in the rainy season at first antenatal care visit raises to 60 % [3]. Placental malaria (PM) has been reported up to 40 % at delivery [4]. Infected red blood cells present at their surface the *P.falciparum* erythrocyte membrane protein1 (PfEMP1) expressed by the parasite and coded by the var gene family. There are different types of this protein binding to various receptors on the endothelium of the blood vessels [2]. Literature on placental malaria indicates mostly that chondroitin sulfate A (CSA) is the receptor in the placenta on which bind the parasitized red blood cells presenting the variant VAR2CSA a subgroup of the PfEMP1 [2, 5]. This allows the parasites to sequestrate and accumulate in the intervillous spaces [6]. Hyaluronic acid was cited as another receptor of *P. falciparum[5]*. However, the results are unclear as its activity seemed to be mediated through chondroitin sulfate [5]. Placental malaria can result in maternal anemia [7], low birth weight (LBW), or preterm delivery (PTD) [7–9] which can induce neurocognitive and other health disorders later in life [10, 11].

Pregnant women acquire protective immunity against PM as their ages increase. That is why younger pregnant women are more prone to PM than their older counterparts [12, 13]. Also, women pregnant for the first time (primigravidae) are more subject to placental malaria in endemic settings compared to those with second pregnancy or more (multigravidae) [14, 15]. The immune reaction initiated as a response against the infection occurring in the first pregnancy become more and more protective with the subsequent pregnancies as antibodies are developed against the parasite and prevent it binding and sequestrating into the placenta [12, 16].

We hypothesized that the immunity related to age may influence the relationship between gravidity and other maternal characteristics and PM. While factors associated with placental malaria have been studied widely, there is little information on these potential interactions [9, 15, 17–22]. Few studies assessed factors associated with PM in Burkina Faso where malaria is endemic with seasonal transmission [15, 23]. In a study conducted in Nanoro in 2013, approximately 43 % of pregnant women attending antenatal care visits presented with peripheral parasitemia and therefore were at increased risk of PM [24].

The current study aims to assess the prevalence of PM in Burkina Faso and to assess if factors associated with PM are depending on age.

## Methods

### Study settings and population

The present study used data collected in the framework of a multicenter, randomized, open-label trial of treatments for malaria in pregnant women entitled: “safe and efficacious artemisinin-based combination treatments for African pregnant women with malaria (PREGACT)” which is registered under the number NCT00852423. The latter evaluated the efficacy and safety of four artemisinin-based combination treatments (ACT) in pregnant women with *P. falciparum* parasitemia whatever the presence of symptoms. However, women with symptoms and/or signs (clinical or biological) of severe malaria were not included. Detailed methodology and study results were published elsewhere [25, 26].

### Recruitment and follow-up

Briefly, second or thirdtrimester pregnant women with microscopically confirmed *P. falciparum* peripheral infection, who were willing to give informed consent, were enrolled and treated with one of the four ACTs at random and followed up until delivery. All participants were provided a long-lasting insecticidal bed net upon inclusion.

### Data collection

At inclusion in the parent study, obstetrical, medical, demographic, and anthropometric data were recorded. The use of malaria prevention methods before the inclusion in the study was documented. Peripheral malaria infection was confirmed and parasites density and species were determined by microscopic examination of thick and thin blood smear stained with Giemsa [25]. Hemoglobin level was determined using a portable HemoCue (Hb301 Hemocue®, Angelholm, Sweden). Gestational age and fetal viability were confirmed ultrasonographically using a portable Fukuda Denshi® machine (FFsonic, UF – 4100).

The cure of the initial episode was ascertained on the basis of a negative blood smear obtained at the end of the three days treatment course. Peripheral malaria parasitemia occurring after the this episodeuntil delivery was recorded.

Delivery date and mode, gestational age, and newborn characteristics were collected. Also, a biopsy of the placenta was collected.

### Placental malaria diagnosis

Biopsies pieces were collected from the maternal side of the placenta, as soon as possible after delivery and kept in a neutral buffered 10 % formalin. Subsequent procedures comprised embedment in paraffin wax, slicing in 4 mm thick sections, and staining with hematoxylin– eosin. Reading and classification of PM patterns were done according to the method described by Ismail. Acute PM infection was considered in presence of parasites and absence of malaria pigment. Chronic PM infection was considered in presence of both malaria parasites and malaria pigment. A past PM was considered in absence of malaria parasites but the presence of malaria pigment. No PM infection was considered when there were no malaria parasites nor malaria pigment [27]. An external quality control (EQC) was performed during the study. Before the start of the study, a training set of placental slides prepared by the EQC center was sent to the site for evaluation. At the start of the study, the first 25 samples included in the study site were sent to the EQC center for revision. Finally, 5 % of the samples were revised by the EQC center. Active PM comprised acute or chronic placental infections and represented all placentas with parasites, while past-chronic PM referred to past or chronic infections, corresponding to all placentas with malaria pigment. Gravidity did not include the present pregnancy. Symptomatic malaria referred to , fever at enrolment with parasitemia regardless of the density, or parasitemia witha minimum of three of the following symptoms: fever in the past 24 hours, muscular- and articular pain, head pain, tiredness or weakness [26]. Recurrent malaria was defined as the occurrence of a malaria infection after initial treatment and before delivery regardless of the number of episodes. Anemia was defined as a hemoglobin level lower than 11g/dl. Underweight was defined as pregnancy body mass index < 18.50 kg/m^2^. Preterm birth was defined as a birth occurring before 37 weeks of gestation and low birth weight was defined as birth weight below 2500 g. The season at delivery was either rainy (July to December) or dry (January to June) [15].

### Statistical analysis

The gestational age at delivery was calculated from the estimation done by ultrasonography at inclusion. The association of placental malaria with maternal characteristics was assessed using logistic regression. Only factors associated with PM with p-value < 0.05 in the univariate analysis were kept for the multivariate models. The modifying effect of age was assessed by introducing interactions in logistic regression. Interactions of age with the following maternal characteristics were considered: gravidity, anemia, symptomatic malaria at study start, recurrent malaria during follow-up, and season at delivery. When an interaction was statistically significant, the odds ratios of the association of these variables with placental malaria and their 95 % confidence intervals were estimated by age category. Outcome variables were either pooled PM, active PM, or past-chronic PM. Distribution of low birth weight (LBW) and prematurity by placental malaria status was also outlined.

The statistical significance level was set at 0.05. All analyses were performed using

STATA® statistical software version 16 (Statacorp LLC, Texas, USA).

Only one woman was pregnant for the first time and this did not allow us to create a separate group of primigravidae.

### Ethics

The PREGACT study was approved by the ethics committee of the University of Antwerp in Belgium and the National Ethics Committee of Burkina Faso. Written or witnessed thumb-printed informed consent was obtained from all participants before inclusion and any study procedure was undertaken. All the procedures of the study were carried out according to the guidelines of the ethics committees of Burkina Faso and Belgium that provided approvals.

## Results

### Characteristics of study participants

**Fig. 1:**
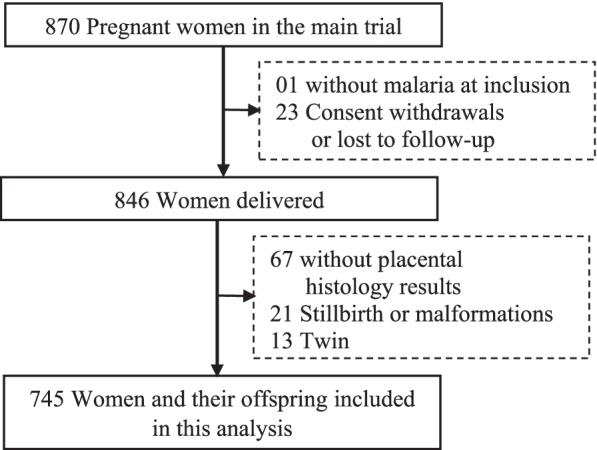
Flow-chart of the pregnant women and their newborns included

From June 2010 to April 2013, 870 pregnant women in their second or third trimester were recruited into the PREGACT trial at Nanoro and Nazoanga sites. Among the 847 women who delivered, 779 had a placental histology assessment. After excluding 13 twins and 21 stillbirths or malformations, the remaining cohort included 745 participants (Fig. 1).

At inclusion, 588 (78.9 %) women were under 30 years, 355 (47.7 %) had less than 3 previous pregnancies, 308 (41.4 %) had symptomatic malaria, and 447 (74.2 %) had anemia (Table 1).

**Table 1:** Mother baseline, follow – up and newborn characteristics

Mother baseline	
Age (years)	
Mean ± sd	24 ± 6
Median (IQR)	23 (20 – 29)
< 30 years	588 (78.9)
Gravidity	
Median(IQR)	3 (1 – 4)
≤ 2^*^	355 (47.7)
No use of bednet the night before recruitment	370 (49.7)
Did not received IPTp before recruitment	648 (87.0)
Anemia at recruitment (Hb < 11 g/ dl)	553 (74.2)
Underweight at recruitment (BMI< 18.5 kg/m^2^)	46 (6.2)
Symptomatic malaria at recruitment^*^	308 (41.4)
Treatment at random^*^	
AL	242 (32.5)
ASAQ	255 (34.3)
MQAS	247 (33.2)
**Mother follow - up**	
Recurrent malaria during follow-up^*^	289 (38.9)
Delivery in the rainy season	447 (60.1)
**Newborn**	
Girl ^*^	358 (48.1)
Birth weight^*^ (g)	
mean ± sd	2838 ± 439
< 2500g	133 (17.9)
Preterm birth (gestation < 37 weeks)	94 (12.6)
Low birth weight or preterm	175 (23.5)

The numbers are n (%) unless stated

*IQR* interquartile range, *IPTp* intermittent preventive treatment in pregnancy with sulfadoxine-pyrimethamine, *Hb* hemoglobin, *BMI* body mass index, *AL* artemether-lumefantrine, *ASAQ* artesunate-amodiaquine, *MQAS* mefloquine-artesunate, *sd* standard deviation

^*^ Missing data for one participant for gravidity, symptomatic malaria, treatment, newborn gender, birth weight, and for two women for recurrent malaria

### Patterns and distribution of placental malaria

**Fig. 2:**
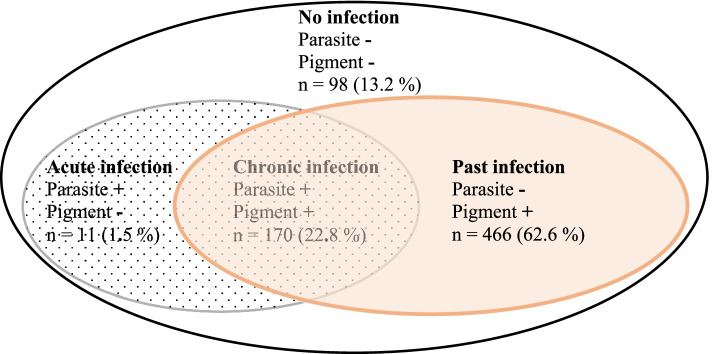
Histopathological diagnosis and distribution of placental malaria. Legend: +: present; -: absent. The region of the grey circle with black dots in the background corresponds to the presence of parasites only in the placenta and represents active placental malaria. The region of the orange circle with orange background corresponds to the presence of malaria pigment only in the placenta and represents past placental malaria. The region where the two circles overlap corresponds to the presence of both parasites and malaria pigment and represents chronic placental malaria. The region of the black circle with white background corresponds to the absence of both parasites or malaria pigment and represents placenta free of malaria infection

Overall, PM was diagnosed in 647 (86.8 %) women. Parasites were seen in 181 (24.3 %) of the placentas and malaria pigment in 636 (85.4 %). Acute, chronic, and past placental malaria was diagnosed in 11 (1.5 %), 170 (22.8 %) and 466 (62.6 %) women, respectively. (Fig. 2). Past infection prevalence was 69.4 % in women delivering in the dry season. Women under 30 years of age experienced more past PM than women of 30 years or over (64.8 % and 54.1 %, respectively). The past PM was more frequent when gravidity ≤ 2 than when gravidity > 2 (65.1% and 60.3%, respectively). Chronic PM distribution followed a trend similar to past PM for maternal age groups (25.9 % in women < 30 years and 11.5 % in women ≥ 30 years) and gravidity groups (31.0 % in women with gravidity ≤ 2 and 15.4 % in women with gravidity > 2). Acute infections were scarce and observed mostly in women of 30 years or more (3.8 %) (Table 2).

**Table 2:** Placental malaria distribution by maternal factors

Maternal Factors	N	Placental malaria			
		No	Past	Chronic	Acute
**Baseline**					
Age (years)					
< 30	588	50 (8.50)	381 (64.8)	152 (25.9)	5 (0.9)
≥ 30	157	48 (30.6)	85 (54.1)	18 (11.5)	6 (3.8)
Gravidity					
≤ 2	355	12 (3.4)	231 (65.1)	110 (31.0)	2 (0.6)
> 2	390	86 (22.1)	235 (60.3)	60 (15.4)	9 (2.3)
Bednet use the previous night					
No	370	46 (12.4)	215 (58.1)	105 (28.4)	4 (1.1)
Yes	375	52 (13.9)	251 (66.9)	65 (17.3)	7 (1.9)
Received IPTp before study start					
Yes	97	8 (8.2)	68 (70.1)	17 (17.5)	4 (4.1)
No	648	90 (13.9)	398 (61.4)	153 (23.6)	7 (1.1)
Anemia at study start(Hb <11g/dl)					
Yes	553	61 (11.0)	349 (63.1)	137 (24.8)	6 (1.1)
No	192	37 (19.3)	117 (60.9)	33 (17.2)	5 (2.6)
Underweight at study start(BMI <18.5kg/m^2^)					
Yes	46	3 (6.5)	30 (65.2)	13 (28.3)	0 (0.0)
No	699	95 (13.6)	436 (62.4)	157 (22.5)	11 (1.6)
Symptomatic malaria at study start*					
Yes	308	24 (7.8)	191 (62.0)	91 (29.5)	2 (0.6)
No	436	74 (17.0)	275 (63.1)	78 (17.9)	9 (2.1)
Treatment at random*					
AL	242	29 (12.0)	159 (65.7)	50 (20.7)	4 (1.7)
ASAQ	255	31 (12.2)	161 (63.1)	61 (23.9)	2 (0.8)
MQAS	247	38 (15.4)	146 (59.1)	58 (23.5)	5 (2.0)
**Follow-up**					
Recurrent malaria during follow-up^*^					
Yes	289	19 (6.6)	172 (59.5)	94 (32.5)	4 (1.4)
No	454	79 (17.4)	293 (64.5)	75 (16.5)	7 (1.5)
Season at delivery^*^					
Rainy	447	35 (7.8)	259 (57.9)	144 (32.2)	9 (2.0)
Dry	297	63 (21.2)	206 (69.4)	26 (8.8)	2 (0.7)

Numbers are frequencies (percentages)

*IPTp* intermittent preventive treatment of malaria during pregnancy with sulfadoxine-pyrimethamine, *AL* artemether-lumefantrine, *ASAQ* artesunate-amodiaquine, *MQAS* mefloquine-artesunate, *Hb* hemoglobin, *BMI* body mass index

*Missing data for one woman for symptomatic malaria, treatment at random, season at delivery and for two women for recurrent malaria.

### Factors associated with placental malaria

**Fig. 3:**
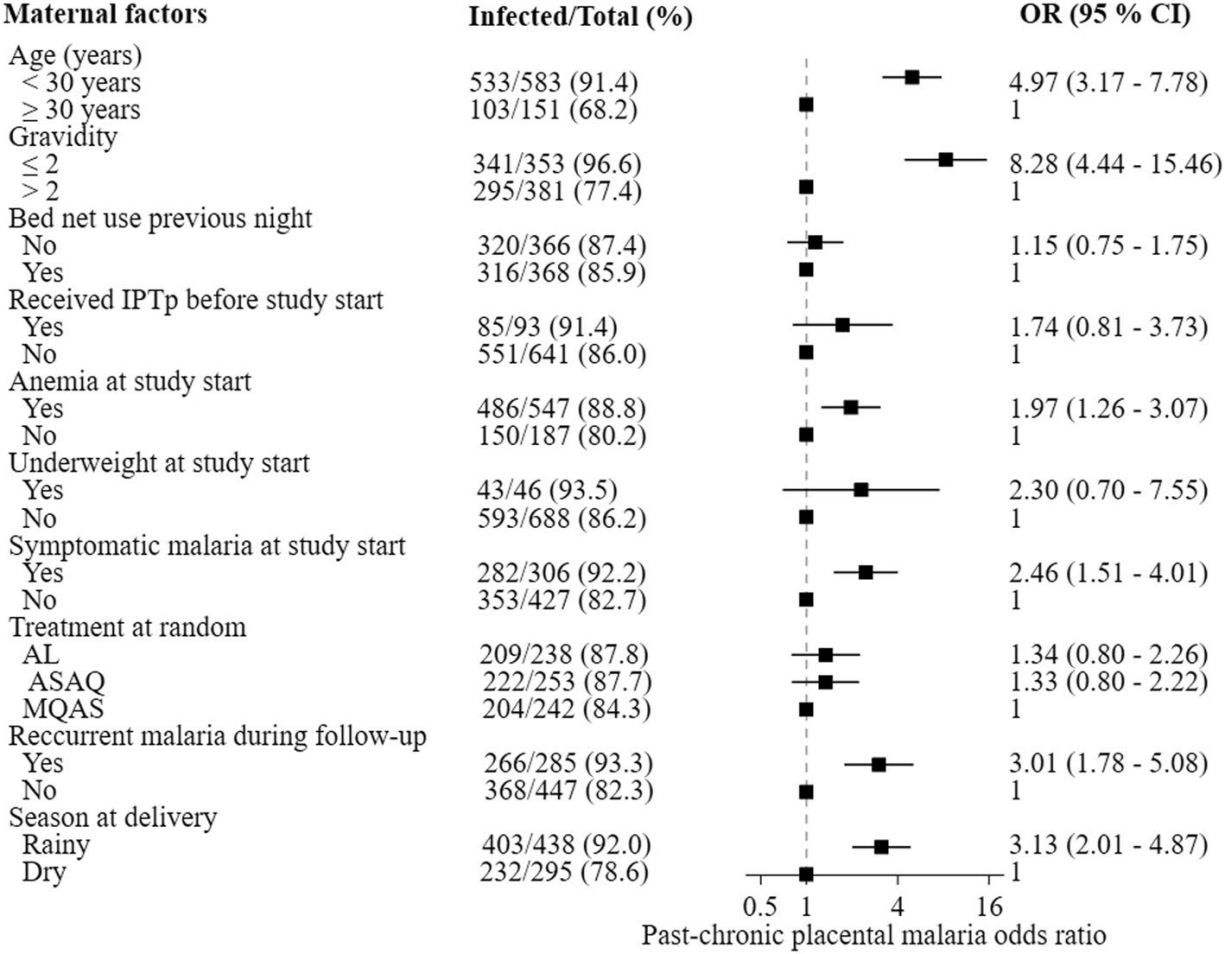
Maternal factors associated with past-chronic placental malaria in univariate analysis. Legend: OR: odds ratio; CI: confidence interval; IPTp: intermittent preventive treatment during pregnancy with sulfadoxine-pyrimethamine; AL; artemether-lumefantrine; ASAQ: artesunate-amodiaquine; MQAS: mefloquine-artesunate. Anemia: hemoglobin < 11g/dl; Underweight: body mass index < 18.5 kg/m^2^; Rainy season: July-December; Dry season: January-June

**Fig. 4:**
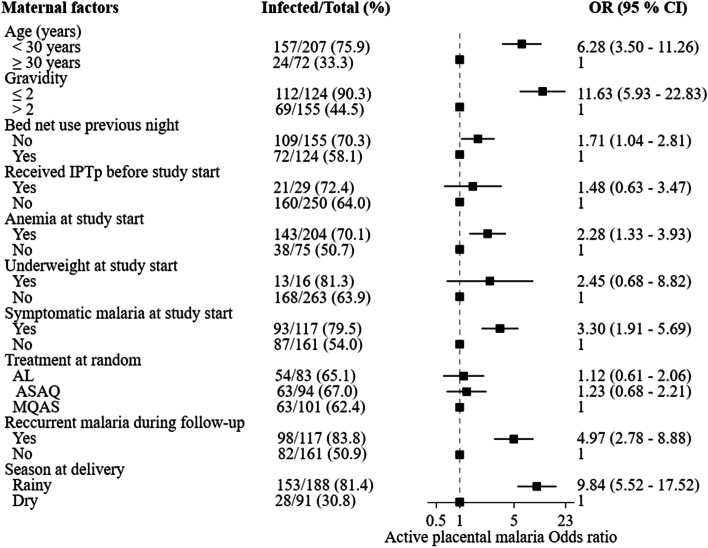
Maternal factors associated with active placental malaria in univariate analysis. Legend: OR: odds ratio; CI: confidence interval; IPTp: intermittent preventive treatment during pregnancy with sulfadoxine-pyrimethamine; AL; artemether-lumefantrine; ASAQ: artesunate-amodiaquine; MQAS: mefloquine-artesunate. Anemia: hemoglobin < 11g/dl; Underweight: body mass index < 18.5 kg/m^2^; Rainy season: July-December; Dry season: January-June

In univariate analysis, age < 30 years, gravidity ≤ 2, anemia, symptomatic malaria at inclusion, recurrent malaria during follow-up, and rainy season at delivery were associated with pooled PM (Table 3), with past-chronic PM (Fig. 3), and with active PM (Fig. 4). Women who did not sleep under a bed net the night before their enrollment into the main trial had a higher prevalence of active PM than women who slept under a bed net.

**Table 3:** Maternal factors associated with pooled placental malaria in univariate analysis

Factors	Pooled placental^*^ malaria [n / N (%)]	OR (95 % CI)
Age (years)		
< 30	538 / 588 (91.5)	4.74 (3.03 - 7.40)
≥ 30	109 / 157 (69.4)	1
Gravidity^†^		
≤ 2	343 / 355 (96.6)	8.09 (4.34 - 15.08)
> 2	304 / 390 (77.9)	1
Bednet use the previous night		
Not used	324 / 370 (87.6)	1.13 (0.74 – 1.74)
Used	323 / 375 (86.1)	1
Received IPTp before study start		
Yes	89 / 97 (91.8)	1.79 (0.84 - 3.82)
No	558 / 648 (86.1)	1
Anemia at study start (Hb < 11g/dl)		
Yes	492 / 553 (89.0)	1.93 (1.23 - 3.01)
No	155 / 192 (80.7)	1
Underweight at study start (BMI <18.5kg/m^2^)		
Yes	43 / 46 (93.5)	2.25 (0.69 - 7.41)
No	604 / 699 (86.4)	1
Symptomatic malaria at study start^†^		
Yes	284 / 308 (92.2)	2.42 (1.49 - 3.93)
No	362 / 436 (83.0)	1
Treatment at random^†^		
AL	213 / 242 (88.0)	1.34 (0.79 - 2.25)
ASAQ	224 / 255 (87.8)	1.31 (0.79 - 2.19)
MQAS	209 / 247 (84.6)	1
Recurrent malaria during follow-up^†^		
Yes	270 / 289 (93.4)	2.99 (1.77 - 5.06)
No	375 / 454 (82.6)	1
Season at delivery		
Rainy	412 / 447 (92.2)	3.17 (2.03 – 4.94)
Dry	234 / 297 (78.8)	1

*OR* odds ratio, *CI* confidence interval, *IPTp* intermittent preventive treatment in pregnancy, *AL* artemether-lumefantrine, *ASAQ* artesunate-amodiaquine, *MQAS* mefloquine – artesunate, *Hb* hemoglobin, *BMI* body mass index.* Pooled placental malaria correspond to the presence of parasites and/or pigment in the placental.^†^ missing data for one participant for symptomatic malaria at study start, treatment, and for two women for recurrent malaria

In multivariable analysis, there was a modifying effect in the association of maternal characteristics with PM. In women under 30 years of age, gravidity ≤ 2 was associated with a higher prevalence of pooled PM but in women of 30 years or more, gravidity was no more associated with pooled PM (OR 6.81, 95 % CI 3.18 – 14.60; and OR 0.52, 95 % CI 0.10 – 2.76, respectively). Anemia at enrollment was also associated with pooled PM in women under 30 years of age (OR 1.96, 95 % CI 1.03 – 3.72) but not in women of 30 years or more (OR 0.68, 95 % CI 0.31 – 1.49) (Table 4). A modifying effect was also observed in the association of gravidity with past-chronic PM (Table 5). Symptomatic malaria was associated with active PM in women under 30 years of age (OR 3.79, 95 % CI 1.55 – 9.27). The association was not significant in women of 30 years or more (OR 0.42, 95 % CI 0.10 – 1.75) (Table 6).

**Table 4:** Maternal factors associated with pooled placental malaria in multivariable analysis

Factors	OR (95 % CI)
Recurrent malaria during follow-up	
Yes	2.02 (1.15 – 3.55)
No	1
Season at delivery	
Rainy	3.24 (1.99 – 5.29)
Dry	1
Age < 30 years	
Gravidity ≤ 2	6.81 (3.18 – 14.60)
Gravidity > 2	1
Age ≥ 30 years	
Gravidity ≤ 2	0.52 (0.10 – 2.76)
Gravidity > 2	1
Age < 30 years	
Anemia at study start	1.96 (1.03 – 3.72)
No anemia at study start	1
Age ≥ 30 years	
Anemia at study start	0.68 (0.31 – 1.49)
No anemia at study start	1

*OR* odds ratios, *CI* confidence interval

**Table 5:** Maternal factors associated with past-chronic placental malaria in multivariable analysis

Factors	OR (95 % CI)
Recurrent malaria during follow-up	
Yes	2.01 (1.15 – 3.54)
No	1
Season at delivery	
Rainy	3.16 (1.94 – 5.13)
Dry	1
Age < 30 years	
Gravidity ≤ 2	7.74 (3.65 – 16.44)
Gravidity > 2	1
Age ≥ 30 years	
Gravidity ≤ 2	0.46 (0.08 – 2.72)
Gravidity > 2	1

*OR* odds ratios, *CI* confidence interval, *Hb* hemoglobin

**Table 6:** Maternal factors associated with active placental malaria in multivariable analysis

Factors	OR (95 % CI)
Recurrent malaria	
Yes	2.77 (1.35 – 5.70)
No	1
Delivery in the rainy season	
Yes	6.92 (3.38 – 14.14)
No	1
Gravidity	
≤ 2	6.01 (2.62 – 13.80)
> 2	1
Age < 30 years	
Symptomatic malaria	3.79 (1.55 – 9.27)
Asymptomatic malaria	1
Age ≥ 30 years	
Symptomatic malaria	0.42 (0.10 – 1.75)
Asymptomatic malaria	1

*OR* odds ratio, *CI* confidence interval

### Distribution of low birth weight or prematurity by placental malaria patterns

**Fig. 5:**
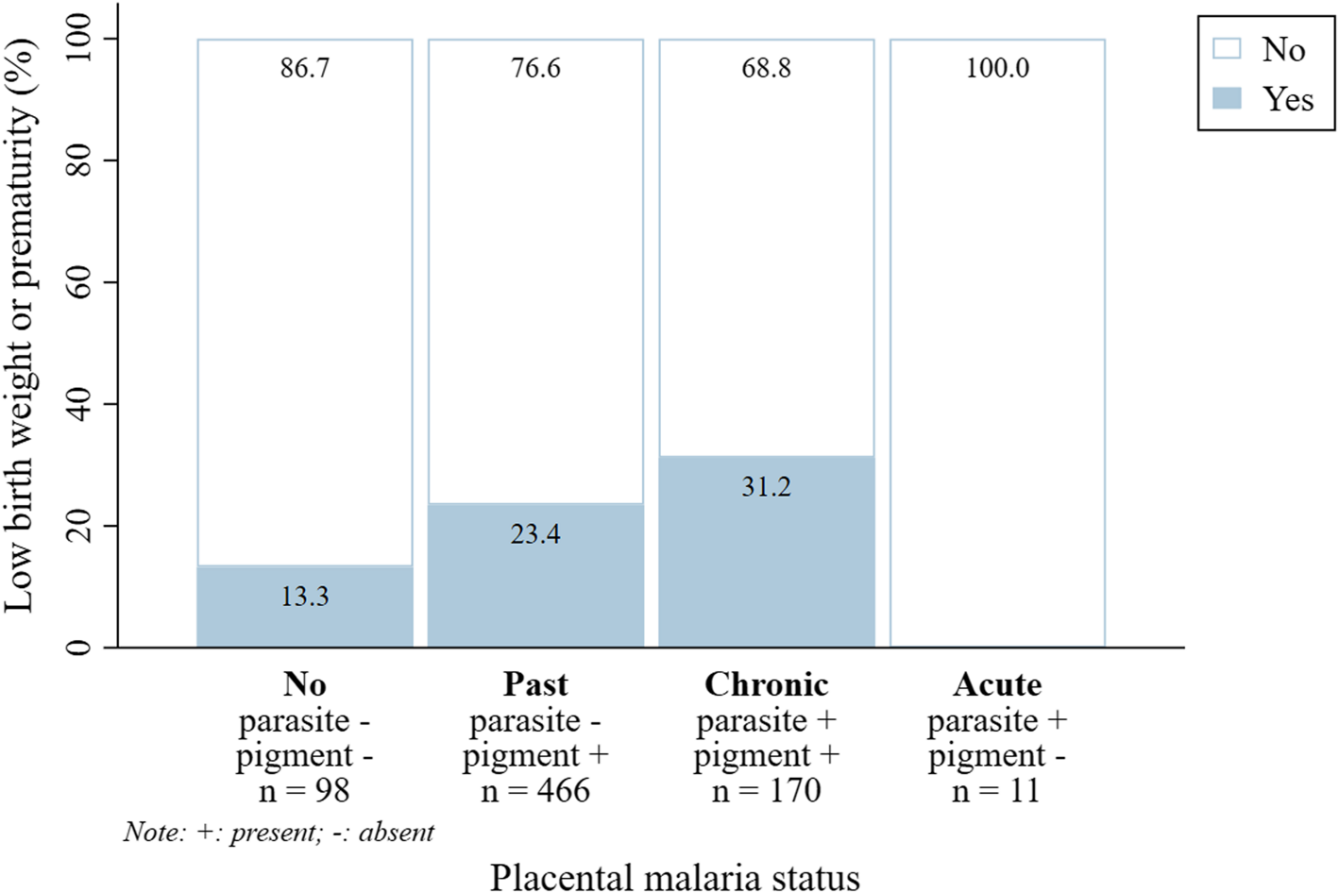
Low birth weight and/or prematurity by placental malaria status. Legend: +: present; -: absent

LBW or prematurity was observed in 31.2 % and 23.4 % of newborns from mothers with chronic PM and past PM, respectively. Among newborns from women without PM, 13.3 % were LBW or preterm. Surprisingly, none of the newborns from mothers with acute PM was LBW or preterm. (Fig. 5).

## Discussion

Our study showed that PM prevalence was high in this cohort of pregnant women with *P. falciparum* peripheral infection at inclusion. Past infections were the predominant PM pattern, followed by chronic infections, acute infections being scarce. A modifying effect according to age was noticed in the association of gravidity, anemia, and symptomatic malaria with PM. Our data also evidenced a higher prevalence of LBW or prematurity in newborns from mothers with chronic PM compared to newborns from mothers with past PM or without PM infection.

We reported 86.8 % of women with PM. Placental malaria is expected to be more prevalent in mothers with peripheral infection than in mothers without detectable peripheral infection, as reported in Malawi, in India, and a metanalysis [20, 28, 29]. The lower PM prevalence in Malawi (78.3%) compared to our study could be explained by the peri-urban characteristic of the Malawi study location. Also, malaria in pregnancy burden is usually higher in rural areas like in our study setting than in urban areas [30]. Peripheral infections in our study were all due to *P. falciparum,* the malaria parasite with the greatest potential to accumulate in the placenta [31]. We hypothesized that this contributed to the higher prevalence of PM in our study compared to an Indian study (64.1 %) where mixed *P.falciparum* and *Plasmodium vivax* peripheral infections occurred as well [20].

A lower proportion (56 % to 58 %) of infected placentas compared to our study were reported in the same area in another multicentric study. This difference could be explained by the inclusion of both women with and without peripheral infection in the latter [15].

Malaria parasites were found in 24.3 % (active infection) of the placentas in our study, while malaria pigment was present in 85.4 %. Malaria pigment could be a marker of peripheral parasitemia at the initial stage of the pregnancy, active infection reflecting more near-term intense peripheral infection [29, 32]. It was also suggested that without treatment, malaria pigment could be a marker of prior infection of the placenta [33].

In our study, 13.2 % of the placentas were uninfected. Such discordance between peripheral and placental malaria was already reported in Nigeria. Authors attributed this to peripheral parasitemia of low density occurring at the beginning of the pregnancy [19], probably cleared by chemoprevention [15] before reaching the placenta. It is reported that with time, malaria pigment can be cleaned from the placenta following a rapid and efficacious treatment. Artemether-lumefantrine (AL) has even been found associated with less accumulation of malaria pigment in the placenta than quinine [34–36]. In our study, all malaria peripheral infections (initial and subsequent) in the study participants were treated with highly efficacious artemisinin-based combination therapies including AL, regardless of the density of the parasites or the presence of symptoms [26]. This probably contributed to prevent or clear placental malaria in a small proportion of them.

Maternal age [7, 37, 38], gravidity [15, 18, 39, 40], anemia [9, 28], and symptoms [9, 41] are some of the factors associated with placental malaria in studies conducted in pregnant women regardless of patent peripheral parasitemia. The above-mentioned factors were also associated with the presence of malaria pigment in a metanalysis conducted in 2020 using data from a quite selected population of pregnant women with peripheral parasitemia at enrolment.

The association of gravidity, anemia at study start, and symptomatic malaria at study start with PM was modified by maternal age in our study.

Placental malaria affects primigravidae or secundigravidae more than multigravidae [9, 15, 18, 40]. In our study, pooled PM and past-chronic PM were more frequent in younger women with lower gravidity. These findings suggest that for a certain pattern of PM, the protective effect of parity-specific immunity depends on maternal age [12]. Indeed, a study modeling the immunity against placental malaria-specific to the parity in 2013 showed that the highest level of this immunity was overloaded when the entomological inoculation rate (EIR) exceeds 100 [33]. As EIR is usually above that threshold in most of the countries where malaria transmission is high and stable [42], this suggests that the risk of placental malaria might be similar in pregnant women whatever their gravidities in such settings. This is true for older women in our study while younger women with lower gravidities remained more at risk of placental malaria. Thus, this supposes that in settings where malaria transmission is high and stable, age-related immunity contributes significantly to the control of the infection as far as immunity related to parity [12].

In our study, a higher prevalence of pooled PM was observed in anemic women aged less than 30 years. Infected red blood cells bind to the vascular walls in the bone marrow. This results in a lower production of red blood cells contributing to anemia [43, 44]. In malaria-endemic regions, older pregnant women acquire antibodies that prevent infected red blood cells from binding to the vascular walls [2, 45]. We infer that younger pregnant women are producing less protective antibodies and this is why they are more prone to both anemia and PM [2, 30].

Undernutrition is one of the main causes of anemia through iron insufficiency in low and middle-income countries [46, 47] like Burkina Faso. Studies conducted in several countries across sub-Saharan Africa reported a higher prevalence of anemia in younger pregnant women than in older [48–50] irrespective of malaria infection. However, there is a probable interaction between malaria and undernutrition [51]. As the nutritional status influences immunity, the ability of an individual to regulate and eradicate infection like malaria could be modified by undernutrition [52]. Conversely, the proteins are increasingly destroyed and the energy consumed during the inflammatory response induced by an infection like malaria. This could lead to the depletion of the nutritional reserve [51, 53]. We hypothesized that the increased prevalence of placental malaria in young anemic women in our study might illustrate the interplay between malaria and nutrition.

Pregnant women could benefit from iron and folic acid supplements without being exposed to an increased risk of malaria compared to those receiving folic acid only [54]. However, the benefit of this supplementation for their offspring was questionable as a study conducted at the Nanoro site reported an increased risk of prematurity in women supplemented with iron and folic acid compared to women supplemented with folic acid alone [55].

Symptomatic malaria was associated with active PM in women aged less than 30 years of age in our study. Acquired immunity against malaria is weak in younger women and may be compared to that of women residing in regions. As a result, they may experience more clinical episodes [13, 56].

Fever is the most common symptom in individuals infected by malaria. The presence of fever implies intense parasitemia above the pyrogenic threshold overpassing the tolerance capacity [57–59]. This suggests that in young symptomatic women the number of parasites reaching the placenta is increased because of the high density of parasitemia in the bloodstream. Some authors proposed to use symptomatic malaria as a proxy for PM diagnosis antenatally [60]. In sub-Saharan malaria-endemic regions, this would underestimate the burden of PM as peripheral infections are often asymptomatic [24].

The risk factors of placental malaria are not limited to the above-mentioned. They include environmental and socio-economic factors as far as the use of preventive measures against malaria. Indeed, placental malaria prevalence was higher in women delivering in the dry season compared to those delivering in the rainy season in a study conducted in Benin, Burkina Faso, and The Gambia [15]. In Cameroon, pregnant women who earned less than 28 000 fcfa per month were more at risk of placental malaria [61]. A low educational level may be associated with an increased risk of placental malaria as found in Nigeria [37]. Pregnant women who did not sleep under bed net were more at risk of placental malaria [62, 63]. In our study, the season of delivery was significantly associated with placental malaria with infections occurring more in the rainy season than the dry season. This association did not depend on age. Bed net use and IPTp-SP were not associated with placental malaria in our multivariate analysis.

[64, 65, 43, 44, 2, 45, 54, 55, 13, 56, 60] Reduced birth weight and preterm birth were more prevalent with chronic infections [9, 66]. In our study, the composite outcome of LBW and prematurity was also more frequent in newborns from mothers with chronic PM. The fetal growth restriction caused by the inflammatory response to chronic infection was cited as a possible contributing factor of LBW [8]. These adverse pregnancy outcomes associated with placental malaria underline the need of protecting pregnant women from malaria. Placental malaria incidence could be significantly reduced in endemic areas through the deployment of preventive measures in the first stage of the pregnancy [33]. It was estimated that up to 65 % of placental malaria occurred during the first trimester of pregnancy [67]. As IPTp-SP cannot be given at that time, the WHO recommends starting the first dose as soon as possible in the second trimester [68]. The emergence of parasites strains resisting SP did not yet compromise its efficacy in West African countries like Burkina Faso. The resistance ranged from low to moderate [69], thus SP is still used for IPTp and remains efficacious against asymptomatic parasitemia [70, 71]. Evaluation of alternative drugs for IPTp like dihydroartemisinin-piperaquine or mefloquine is ongoing, as well as exploration of new strategies such as community-based malaria screening and treatment in pregnancy, alone or in combination with IPTp-SP [15, 72, 73]. Placental malaria prevention could be better achieved by adding the benefit from sleeping under an insecticide-treated bed net starting in the first trimester of pregnancy to that of SP intake [69]. Also, it is known that a substantial part of malaria infection detected at the beginning of the pregnancy likely originate in the preconception period [3, 33, 74]. Therefore, some authors suggest extending prevention actions to this period too [3, 33].

The present analysis had some limitations. The study was not designed primarily to assess factors associated with PM. Thus, we did not assess some factors like educational and socio-economic levels. This information was not captured in the database of the parent study.

In the present study, we aimed at exploring the extent to which age could be associated with placental malaria. Thus, we chose 30 years as the cut-off, going beyond the usual cut-off of fewer than 25 years in most of the studies investigating the relationship between placental malaria and age [7]. This could seem arbitrary. However, our cut-off is close to the mean age of childbearing of 29 years estimated for 2015-2020 for Africa [75]. The cut-off of 30 years was already used in a study assessing risk factors of placental malaria in Nigeria in 2012 [19]. Another study reported a lower utilization of long-lasting insecticidal bed net in women of childbearing age under 30 years than in older women [76]. We explore whether this might represent an extra risk of placental malaria in our cohort. We did not find a significant interaction between age and bed net use before the inclusion in the relationship to placental malaria in multivariable analysis. Only one participant was pregnant for the first time and this may be influenced our results as primigravidae have a different immunity status compared to the others [33]. The study was conducted in a rural area where malaria is highly prevalent compared to urban areas [30]. Therefore, the inference should be limited to such settings. Also, our cohort consisted entirely of women with peripheral infection at enrollment. Therefore, comparison to unselected women could be biased.

However, our study has several strengths. The participants were recruited at peripheral health centers, the first level of contact with the health system and this reduces the chances of a selection bias. The prospective design was also an advantage. Analysis exploring interactions of age with other maternal factors are more relevant to understanding how factors are associated with PM.

## Conclusion

Despite the rapid diagnosis and efficacious treatment of peripheral infections, the prevalence of placental malaria remained high in women with peripheral *P. falciparum* infection in Nanoro, especially in younger women . This underlines the importance of preventive measures in this specific group.

## Data Availability

The datasets supporting these analyses were not publicly available due to ethical and privacy considerations. However, access could be granted upon motivated and reasonable request addressed to Tinto Halidou, e-mail: tintohalidou@gmail.com.

## Abbreviations

°C: Celsius degree
ACT: Artemisinin based combination therapy
AL: Artemether-Lumefantrine
ASAQ: Artesunate-Amodiaquine
CI: Confidence interval
EIR: Entomological inoculation rate
EQC: External quality control
G: Gram
IPTp-SP: Intermittent preventive treatment during pregnancy with sulfadoxine-pyrimethamine
Kg: Kilogramm
LBW: Low birth weight
m^2^: meter square
mm^3^: cubic millimetre
MQAS: Mefloquine-Artesunate
OR: Odds ratio
PM: Placental malaria
PREGACT: Safe and efficacious artemisinin-based combination treatments for African pregnant women with malaria
PTD: Preterm delivery
SP: Sulfadoxine-Pyrimethamine
